# Increased Plasma Heparanase Activity and Endothelial Glycocalyx Degradation in Dengue Patients Is Associated With Plasma Leakage

**DOI:** 10.3389/fimmu.2021.759570

**Published:** 2021-12-20

**Authors:** Baranca Buijsers, Fadel Muhammad Garishah, Silvita Fitri Riswari, Rosalie M. van Ast, Setyo Gundi Pramudo, Rahajeng N. Tunjungputri, Gijs J. Overheul, Ronald P. van Rij, André van der Ven, Bachti Alisjahbana, Muhammad Hussein Gasem, Quirijn de Mast, Johan van der Vlag

**Affiliations:** ^1^ Department of Nephrology, Radboud Institute for Molecular Life Sciences, Radboud University Medical Center, Nijmegen, Netherlands; ^2^ Department of Internal Medicine and the Radboud Center for Infectious Diseases, Radboud University Medical Center, Nijmegen, Netherlands; ^3^ Center for Tropical and Infectious Diseases (CENTRID), Faculty of Medicine, Diponegoro University, Dr. Kariadi Hospital, Semarang, Indonesia; ^4^ Research Center for Care and Control of Infectious Disease (RC3ID), Universitas Padjadjaran, Bandung, Indonesia; ^5^ Department of Biomedical Sciences, Faculty of Medicine, Universitas Padjadjaran, Bandung, Indonesia; ^6^ Department of Internal Medicine, Diponegoro National University Hospital, Faculty of Medicine, Diponegoro University, Semarang, Indonesia; ^7^ Department of Internal Medicine, William Booth Hospital, Semarang, Indonesia; ^8^ Department of Medical Microbiology, Radboud Institute for Molecular Life Sciences, Radboud University Medical Center, Nijmegen, Netherlands; ^9^ Department of Internal Medicine, Hasan Sadikin General Hospital, Faculty of Medicine, Universitas Padjadjaran, Bandung, Indonesia

**Keywords:** dengue, platelets, heparanase, endothelial glycocalyx degradation, vascular permeability syndrome, DENV2

## Abstract

**Background:**

Endothelial hyper-permeability with plasma leakage and thrombocytopenia are predominant features of severe dengue virus infection. It is well established that heparanase, the endothelial glycocalyx degrading enzyme, plays a major role in various diseases with vascular leakage. It is yet to be elucidated whether heparanase activity plays a major role in dengue-associated plasma leakage. Moreover, the major source of heparanase secretion and activation in dengue remains elusive. Since a relatively high amount of heparanase is stored in platelets, we postulate that heparanase released by activated platelets contributes to the increased plasma heparanase activity during dengue virus infection.

**Methods:**

Heparanase activity (plasma and urine), and heparan sulfate and syndecan-1 (plasma levels) were measured in dengue patients with thrombocytopenia in acute phase (n=30), during course of disease (n=10) and in convalescent phase (n=25). Associations with clinical parameters and plasma leakage markers were explored. Platelets from healthy donors were stimulated with dengue non-structural protein-1, DENV2 virus and thrombin to evaluate heparanase release and activity *ex vivo*.

**Results:**

Heparanase activity was elevated in acute dengue and normalized during convalescence. Similarly, glycocalyx components, such as heparan sulfate and syndecan-1, were increased in acute dengue and restored during convalescence. Increased heparanase activity correlated with the endothelial dysfunction markers heparan sulfate and syndecan-1, as well as clinical markers of plasma leakage such as ascites, hematocrit concentration and gall-bladder wall thickening. Notably, platelet number inversely correlated with heparanase activity. *Ex vivo* incubation of platelets with thrombin and live DENV2 virus, but not dengue virus-2-derived non-structural protein 1 induced heparanase release from platelets.

**Conclusion:**

Taken together, our findings suggest that the increase of heparanase activity in dengue patients is associated with endothelial glycocalyx degradation and plasma leakage. Furthermore, thrombin or DENV2 activated platelets may be considered as a potential source of heparanase.

## Introduction

Dengue virus (DENV) infection is the most prevalent arboviral infection in humans globally (1, 2). In 2019, the WHO declared dengue to be one of the top ten threats to global health. In most cases, dengue remains asymptomatic or manifests as an undifferentiated fever. The most important complication is a transient endothelial hyperpermeability syndrome that manifests as bleeding tendency with extravascular fluid accumulation, which can lead to hypovolemic shock and organ failure (3). The exact pathogenesis of the transient endothelial hyper-permeability remains incompletely understood. Pathological examination revealed that severe plasma leakage in dengue patients occurs with a negligible endothelial damage (4).

Vascular hyperpermeability results in elevated exchange of macromolecules and fluids across the endothelium between the interstitial and intravascular space (5). Notably, a significant regulator of vascular permeability is the carbohydrate layer covering the endothelial cells, which is called the endothelial glycocalyx layer (EGL) (6). The EGL consists of glycoproteins with acidic oligosaccharides and terminal sialic acids, as well as membrane-bound proteoglycans, such as syndecan-1, associated with glycosaminoglycan (GAG) side chains. The main sulphated GAG in the endothelial glycocalyx is heparan sulphate (HS) (7). The increased permeability of the endothelium often correlates with degradation of the EGL (8, 9), and shedding of the glycocalyx is strongly associated with severe plasma leakage in dengue cases (10, 11).

Heparanase (HPSE) (12, 13) is the only known human endo-β (1-4)-D-glucuronidase that is able to degrade HS within the endothelial glycocalyx (14). Two recent studies demonstrated the importance of HPSE in the pathogenesis of dengue-associated plasma leakage. Puerta-Guardo et al. showed that endothelial HPSE release can be induced by DENV nonstructural protein-1 (NS1), which is the only highly secreted viral protein from infected host cells, and directly caused disruption of the endothelial barrier function (15). Chen et al. further demonstrated the presence of HPSE protein in the sera of dengue patients (16). Furthermore, HPSE plays a crucial role in compromising the glomerular endothelial barrier function in glomerular disease, which leads to proteinuria (17, 18). Notably, proteinuria has been observed in dengue patients as well, which was associated with disease severity (19). To date, no studies have examined HPSE activity levels in dengue patients and the associations with glycocalyx disruption and plasma leakage.

The cellular origin of HPSE in dengue also remains to be elucidated. Besides the endothelium, various blood cells express HPSE, and in particular platelets abundantly express HPSE, which is released upon platelet activation (20, 21). Platelet activation and thrombocytopenia are well-known features of dengue (22, 23). Platelets are known to play a crucial role in maintaining vascular integrity (24) and modulating leukocyte diapedesis through HPSE secretion (25). In dengue-associated inflammation, platelet activation (22) as well as thrombin generation (26–28) are increased leading to a vicious cycle of thrombin-induced platelet activation. Therefore, we postulate that thrombin and DENV-induced platelet activation might contribute to increased HPSE release and activity in dengue patients.

In this study, we aim to further unravel the role of HPSE in the pathogenesis of dengue. HPSE activity was evaluated in plasma and urine of dengue patients and related to plasma leakage parameters and endothelial glycocalyx degradation products. Furthermore, we assessed the effects of DENV NS1, thrombin, and DENV2 virus in HPSE release from platelets.

## Methods

### Patients and Study Design

This study uses stored citrate plasma samples from 30 adult dengue patients who participated in a placebo controlled randomized clinical trial studying the effect of adjunctive treatment with oseltamivir phosphate on platelet recovery and plasma leakage in adult Indonesian patients with acute dengue virus infection and thrombocytopenia (ISRCTN35227717). Only samples from patients assigned to the placebo arm of the trial are used in the present study. In short, the multi-center trial was performed in Diponegoro National Hospital/Wongsonegoro General Hospital/William Booth General Hospital (Semarang), Kartini General Hospital (Jepara), and Dr. M. Salamun Air Force Hospital/Hasan Sadikin General Hospital (Bandung) in Java, Indonesia between January 2018 and July 2019. Adults hospitalized with suspected dengue with duration of illness ≤ 6 days, thrombocytopenia (<70 x 10^9^ cells/L), and a positive result for rapid DENV NS1 antigen or IgM anti-dengue test (PanBio Diagnostics, Windsor, Australia) were enrolled and followed-up during hospitalization. Blood was collected on a daily basis since inclusion day (D0) until platelet count reached 100x10^9^ cells/L or until hospital discharge, and a convalescent blood sample was collected 3 weeks after inclusion (D21). Plasma leakage was assessed by twice-a-day hematocrit count, daily plasma albumin concentration measurement, and daily assessment of gall-bladder wall thickening and the presence of ascites/pleural effusion using handheld ultrasonography. Adult healthy volunteers (N=10) among students and research staffs in the Department of Internal Medicine and Nephrology, Radboud University Medical Center, Nijmegen, The Netherlands, were included as a control group.

### Ethics Statement

The clinical trial was approved by the Medical Research Ethics Committee, Faculty of Medicine, Diponegoro University, Semarang and Faculty of Medicine, Universitas Padjadjaran, Bandung, Indonesia with reference number No:650/EC/FK-RSDK/XI/2017. Written informed consents were obtained from all subjects.

### Citrate Plasma and Urine Collection and Storage

Citrate plasma samples were obtained from 3.2% citrate-anticoagulated blood (BD Vacutainer, BD Biosciences, USA) centrifuged at 2061g for 15 minutes at room temperature to obtain platelet poor plasma (PPP). Midstream urine samples were collected in the morning. Both plasma and urine samples were stored at -80°C until further analysis.

### Whole Blood Count

Whole blood count was performed from EDTA-anticoagulated blood using standardized automated hematology analyzers in all centers participating in the clinical trial.

### Ultrasonography Measurements

Ultrasonography examination was performed after a minimum 6 hours of fasting in a supine position to measure gall-bladder wall thickness (GBWT) and the presence of ascites and pleural fluid. Sonography examinations were carried out using Phillips Lumify ultrasound with a 5-2 MHz C5-2 transducer.

### HPSE Activity Assay

The activity of HPSE in citrate plasma and urine was measured using a heparan degrading enzyme assay kit (Takara Bio, Shiga, Japan, cat no#MK412) according to the manufacturer’s instruction.

### HPSE Protein Assay

HPSE protein was measured using a human heparanase ELISA kit (Abcam, Cambridge, UK, cat. no. #ab256401) according to the manufacturer’s instruction.

### Syndecan-1 Assay

Syndecan-1 plasma levels were measured using a human syndecan-1 (CD138) ELISA kit (Abcam, Cambridge, UK, cat. no #ab46506) according to manufacturer’s instruction.

### HS Competition Assay

HS in citrate plasma samples was quantified by a previously described HS competition assay (29–31). Briefly, plates were coated overnight in a humidified chamber at room temperature (RT) with 10 μg/ml heparan sulfate from bovine kidney (HSBK) (Sigma-Aldrich, Zwijndrecht, Netherlands) in HS coating buffer. Subsequently, both HSBK coated and uncoated plates were washed with 0.05% PBS-Tween 20 (Sigma-Aldrich) (PBST) and blocked for minimal 2 h with 1% bacto-gelatin (Difco laboratories, Detroit, Michigan, USA) in PBS at RT. Upon blocking, uncoated plates were washed with PBST, whereupon plasma samples were added to the uncoated plate. Plasma samples were four times diluted in PBST containing primary mouse anti-rat IgM HS antibody JM403 (Amsbio, Abingdon, United Kingdom, cat. no. #370730-S, RRID: AB_10890960, final concentration 1.3 μg/ml) and incubated for 1 h at RT. Samples were randomized over plates. Subsequently, the samples were transferred from the uncoated plates to the HSBK-coated plates and incubated for 1 h at RT. Plates were washed with PBST and incubated with secondary goat anti-mouse IgM HRP antibody (Southern Biotech, Uden, Netherlands, cat. no. #1020-05, RRID: AB_2794201, dilution 1:10000 in PBST) for 1 h at RT. Finally, plates were washed with PBST and 3,3’,5,5’-tetramethylbenzidine (TMB) substrate (Invitrogen, Breda, Netherlands) was added and the reaction was stopped by addition of 2 M sulfuric acid, and absorbance was measured at 450 nm. The amount of HS detected in plasma is expressed in arbitrary units, since HS from bovine kidney was coated and used to prepare the standard curve.

### *Ex Vivo* Incubation of Platelets With NS1 or Thrombin

Platelet-rich plasma (PRP) was isolated from healthy donors as previously described (32). In brief, 3.2% citrate-anticoagulated whole blood tubes (BD Vacutainer, Becton Dickinson Biosciences, Franklin Lakes, NJ, USA) were centrifuged at 156g for 15 minutes at room temperature without a break and with slow acceleration. The two third upper layer was considered as platelet-rich plasma and was adjusted to 200 x 10^3^/μL platelets for further stimulations using RPMI 1640 medium (Gibco, Thermo Fisher Scientific, Massachusetts, USA, cat. no. #22409015). Platelets (5, 10 and 20 x10^6^cells/well) were seeded in round-bottomed 96-well plates (Greiner Bio-One, Kremsmünster, Austria, cat. no. #650180) and pre-treated with RPMI or dengue virus-2 non-structural protein-1 (DENV NS1, strain Thailand/16681/84; Native Antigen, Oxford, UK, batch. no. #18042710, final concentration 10 μg/mL) for 20 minutes at 37 °C. Next, platelets were stimulated with thrombin (Synapse Research Institute, Maastricht, The Netherlands, lot. no. #180719, final concentration 0.5U/mL) or RPMI as control for another 20 minutes at 37 °C. Plates were further centrifuged at 430g for 8 minutes to obtain supernatant and stored at -80°C for further analysis.

### DENV2 Virus Culture and Preparation

Mock and DENV serotype 2 (strain 16681) stocks were grown on C6/36 cells in Leibovitz’s L-15 medium (Thermo Fisher Scientific, cat #21083027) supplemented with 10% heat-inactivated fetal bovine serum (FBS) (Sigma-Aldrich, cat #F7524), 2% tryptose phosphate broth (Sigma-Aldrich, cat #T8159), 1× minimum essential media (MEM) non-essential amino acids (Thermo Fisher Scientific, cat #11140035) and 50 U/mL penicillin and 50 µg/mL streptomycin (Thermo Fisher Scientific, cat #15070063) at 28°C without CO_2_. Six days after infection the culture medium was harvested, centrifuged for 5 min at 400g and filtered through an 0.2 μm filter (VWR, cat # 514-0061). Viral titers were measured by end-point dilution on Baby Hamster Kidney 15 (BHK-15) cells, using 10-fold dilutions in 96-well plates and scoring for cytopathic effect (CPE) at 7 days post-infection. BHK-15 cells were cultured in Dulbecco’s Modified Eagle Medium (DMEM), high glucose (Thermo Fisher Scientific, cat #11965092) supplemented with 10% heat-inactivated FBS and 50 U/mL penicillin and 50 µg/mL streptomycin.

### *Ex Vivo* Incubation of Platelets With DENV2 Virus

Platelets (2 x10^6^ cells/well) were seeded in round-bottomed 96-well plates (Greiner Bio-One, Kremsmünster, Austria, cat. no. #650180) and stimulated with RPMI, mock, DENV2 virus ((multiplicity of infection (MOI) 1.6) or thrombin (Synapse Research Institute, Maastricht, The Netherlands, lot. no. #180719, final concentration 0.5 U/mL) for 20 minutes at 37°C. The plates were centrifuged at 430g for 8 minutes and supernatant was stored at -80°C for further analysis.

### Flow Cytometry Analysis of *Ex Vivo* Platelets Incubated With DENV2 NS1, Thrombin or DENV2 Virus

PRP from healthy controls (n=3, method mentioned above) were stimulated with RPMI medium, DENV NS1, thrombin or DENV2 virus as outlined above for 20 minutes at 37 °C. To prevent platelet aggregation, the suspension was supplemented with GPRP (Bachem, Bubendorf, Switzerland, lot. no. #1070771, final concentration 10mM). Upon stimulation, 10 μL of stimulation suspension (2x10^6^ platelets) was added into a mixture of HEPES-buffered saline, saturating concentrations of platelet markers PC7-labelled anti-CD61 antibody (Beckman Coulter, California, USA, cat. no. #IM0540, RRID: AB_2888674, dilution 1:100), PE-labelled anti-CD62p antibody (Bio-Legend, San Diego, USA, cat. no. #304905, RRID: AB_314477, dilution 1:50) and APC-labelled anti-CD107a antibody (Bio-Legend, San Diego, USA, cat. no. #328620, RRID: AB_1279055, dilution 1:50) and incubated for 20 minutes at room temperature in the dark. Subsequently, 0.2% paraformaldehyde solution was added to stop the reaction and platelet activation markers were measured using the CytoFLEX flow cytometer (Beckman Coulter, California, USA). Platelets were gated based on their forward and side scatter plot in a logarithmic scale. Percentage of CD62p and CD107a positive platelets were gated from 10,000 CD61 positive cells. Analyses were further performed using Kaluza software version 2.1 (Beckman Coulter, California, USA).

### Statistical Analyses

Values are expressed as mean ± SEM. D’Agostino & Pearson normality test was performed to test for normality of data. Significance was determined by Chi-square or Fisher’s exact test to compare categorical variables, by Student’s t-test, Wilcoxon-s or Mann Whitney test to compare two groups and by Kruskal-Wallis test followed by Dunn’s test to compare more than two groups. ANOVA or mixed-model analysis followed by Bonferroni/Tukey’s multiple comparison tests were used to assess difference between time point groups. Relationship analysis was performed using Pearson’s or Spearman’s correlation coefficient. All analysis were performed using GraphPad Prism V.8.4.2 (La Jolla, USA). P values less than 0.05 were considered as statistically significant.

## Results

### Characteristics of Study Participants

Clinical characteristics of the patients are summarized in **Table 1**. Most patients were admitted on day 4-6 since fever onset (n=24/30; 80%) and the remaining patients were admitted on day 2-3 since fever onset (n=6/30; 20%). Gall-bladder wall thickness at enrollment median (IQR) value was 3.3 (2.7 – 4.4) mm. At follow up during hospitalization, 11 patients (36.7%) developed ascites, 2 (6.67%) patients had pleural effusion and 3 patients (10%) had both ascites and pleural effusion. Clinical plasma leakage was confirmed in 28 patients (93.3%) using either hemoconcentration, hypoalbuminemia, fluid accumulation and or gall-bladder wall thickening. Hemorrhagic manifestations occurred in 14 patients (46.7%) with dominance of spontaneous petechiae (71.4%).

**Table 1:** Baseline characteristics of patients.

Variables	Acute Dengue
Number	30
Male, n, (%)	21/30 (70)
Age, years, median (IQR)	26 (19 – 33)
Days after fever onset, median (IQR)	5 (4-5)
BMI	21.5 (18-24.3)
NS1 positive, n (%)	19/28 (67.9)
IgM positive, n (%)	14/30 (46.6)
IgG positive, n (%)	12/30 (40)
**Complete blood count at enrollment, median (IQR)**	
Hemoglobin, g/dL	15 (13.2-16.8)
Hematocrit, %	44.3(38.9 – 48.8)
Leukocyte number, x10^9^/L	4.1 (3.3-6.7)
Platelet number, x10^9^/L	44.3 (36-54)
Albumin, g/dL	2.8 (2.6 -3.1)
**Ultrasonography and plasma leakage parameters, n (%)**	
Gall-bladder wall thickening during hospitalization (> 3 mm)	24/30 (80)
Gall-bladder wall thickness at enrollment, median (IQR), mm	3.3 (2.7-4.4)
Ascites or pleural effusion during hospitalization	16/30 (53.3)
Hemoconcentration* during hospitalization	9/30 (30)
Hypoalbuminemia (< 3 g/dL)	24/30 (80)
**Clinical Classification 2009**	
Non-Severe Dengue, n, %	30/30 (100)
**Bleeding manifestations during hospitalization**	**14/30 (46.7)**
Gastrointestinal bleeding, n %	2/14 (14.3)
Hematoma, n %	2/14 (14.3)
Spontaneous petechiae, n %	10/14 (71.4)

Data are presented as median with interquartile range (IQR), number (n) or percentage (%).*Hemoconcentration defined as a single hematocrit value of >50% for male patients or >44% for female patients.

### Plasma and Urine HPSE Activity Is Elevated in Dengue Patients

**Figure 1:**
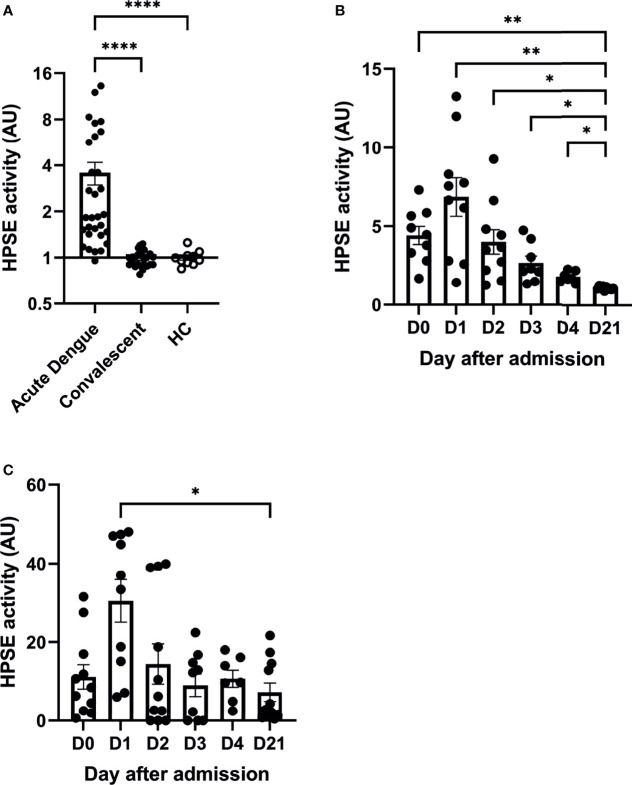
Acute dengue patients display increased plasma and urine HPSE activity. **(A)** HPSE activity in plasma of dengue patients in acute (day 0 or 1 upon hospitalization, n=30) and convalensent (n=25) phase compared to healthy controls (n=10). **(B)** HPSE activity in plasma of acute dengue patients over time (n=10). **(C)** HPSE activity in urine of acute dengue patients over time (n=11). Data were presented as mean ± SEM and tested for normal distribution with D’Agostino & Pearson omnibus normality test. Statistical differences were calculated using mixed-model analysis followed by Bonferroni multiple comparison test or Kruskal-Wallis test followed by Dunn’s multiple comparisons test (*p < 0.05, **p < 0.01, ****p < 0.0001). HPSE, heparanase; HC, healthy controls; AU, arbitrary units; D, day.

Plasma HPSE activity levels in dengue patients at enrollment (n=30) were significantly increased compared to their values in the convalescence phase (n=25; **Figure 1A**). HPSE activity levels in patients in the convalescence phase were similar to healthy controls. Moreover, kinetics of HPSE activity were determined over time during hospitalization in plasma (**Figure 1B**) and urine (**Figure 1C**). HPSE activity was highest one day after study enrollment and decreased in the following days and normalized 21 days after admission (convalescent phase).

### HPSE Activity Correlates With Plasma Leakage and Bleeding Manifestations

**Figure 2:**
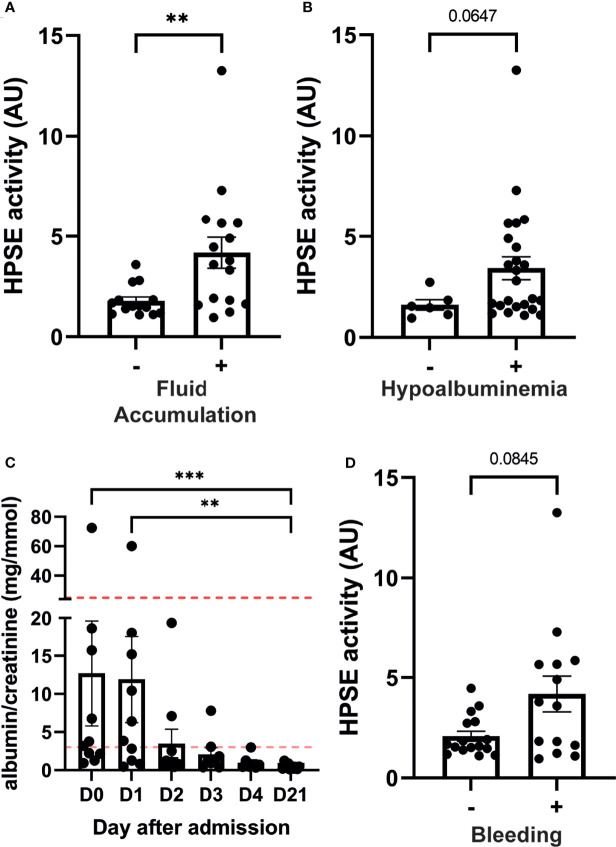
HPSE activity in dengue patients is associated with plasma leakage and bleeding manifestations. Plasma HPSE activity categorised according to the presence of **(A)** Fluid accumulation at enrollment and follow up; Fluid accumulation was determined by the presence of ascites and/or pleural effusion by ultrasonography, **(B)** hypoalbuminemia (plasma albumin level <3 g/dL), and **(C)** bleeding; Bleeding was determined by the presence of one or more bleeding manifestation according to WHO bleeding scale. **(D)** Urinary albumin/creatinine ratio in dengue patients over time (n=10). Horizontal lines indicate microalbuminuria (ACR ≥3.5 mg/mmol (female) or ≥2.5 mg/mmol (male)) and macroalbuminuria (ACR ≥25 mg/mmol (female)). Data were presented as mean ± SEM and distribution normality was assessed with D’Agostino & Pearson omnibus normality test. Statistical differences were calculated using Mann-Whitney U or Kruskal-Wallis test followed by Dunn’s multiple comparisons test (**p < 0.01, ***p < 0.001). HPSE, heparanase; -, in absence of; +, in presence of; AU, arbitrary units; D, day.

Severity of dengue is mostly defined by the presence of fluid accumulation and bleeding manifestations. In our cohort, patients who presented with fluid accumulation (n=16), either ascites or pleural effusion, showed significantly higher plasma heparanase activity (**Figure 2A**). Similarly, patients who displayed hypoalbuminemia tended to have increased HPSE activity (**Figure 2B**). Plasma leakage may result in increases in the hematocrit levels. The hematocrit value showed a strong positive correlation with plasma HPSE activity in acute dengue (**Supplementary Figure 1A**), whereas there tended to be a negative correlation with plasma albumin levels at day 3 (**Supplementary Figure 1B**). Gall-bladder wall thickness, an early marker to assess plasma leakage in dengue patients, showed a positive trend with plasma HPSE activity at day 1 of hospital admission further developing to a strong significant positive correlation at day 2 (**Supplementary Figure 2A**).

The albumin-creatinine ratio (ACR) was determined at enrollment and follow up in ten patients as a marker for glomerular endothelial barrier function. An abnormal ACR [ACR ≥3.5 mg/mmol (female) or ≥2.5 mg/mmol (male)] was detected in six out of ten patients. Interestingly, one patient developed macroalbuminuria (ACR ≥25 mg/mmol (female), which normalized in the convalescent phase (**Figure 2C**). ACR showed a positive trend with urinary HPSE activity at hospitalization further developing to a strong significant positive correlation at day 2 and 3 (**Supplementary Figure 2B**).

Finally, HPSE activity in patients with bleeding manifestations at enrollment or during follow up showed near significant increased levels (p=0.08) compared to patients without bleeding (**Figure 2D**).

### Increased HPSE Activity Is Associated With Increased Endothelial Glycocalyx Degradation Markers in Acute Dengue Virus Infection

**Figure 3:**
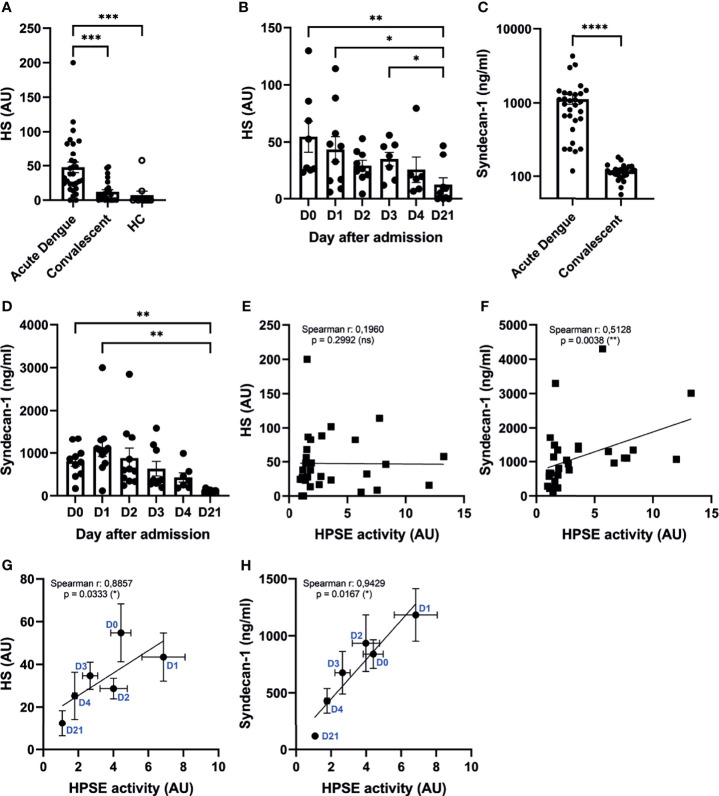
Increased HPSE activity is associated with elevated endothelial glycocalyx degradation markers. **(A)** HS plasma levels in acute dengue (n=30), convalescent dengue (n=25), and healthy controls (n=10). **(B)** HS plasma levels in acute dengue patients over time (n=10). **(C)** Syndecan-1 plasma levels in acute dengue (n=30) and convalescent dengue (n=25). **(D)** Syndecan-1 plasma levels in acute dengue patients over time (n=11). **(E)** Correlation between HPSE activity and HS plasma levels of acute dengue patients at day 1 upon hospitalization (n=30). **(F)** Correlation between HPSE activity and syndecan-1 plasma levels (n=30) in acute dengue patients at day 1 upon hospitalization (n=30). **(G)** Correlation between HPSE activity and HS plasma levels of acute dengue patients over time (n=10). For each time point the mean and SEM HPSE activity/HS levels of all patients measured at that time point is depicted. **(H)** Correlation between HPSE activity and syndecan-1 plasma levels (n=10) in acute dengue patients over time. For each time point the mean and SEM HPSE activity/syndecan-1 levels of all patients measured at that time point is depicted. Data were presented as mean ± SEM and tested for normal distribution with D’Agostino & Pearson omnibus normality test. Statistical differences were calculated using Mann-Whitney U, mixed-model analysis followed by Bonferroni multiple comparison test, Kruskal-Wallis test followed by Dunn’s multiple comparisons test. Correlation analysis was performed with Spearman’s correlation coëfficient (*p < 0.05, **p < 0.01, ***p < 0.001, ****p < 0.0001). HPSE, heparanase; HS, heparan sulfate; AU, arbitrary units; HC, healthy control; D, day.

Besides cleaving HS from the cell surface, HPSE also enhances shedding of transmembrane HS proteoglycan syndecan-1 by upregulating the expression of matrix metalloproteinase 9 (MMP9), which is a syndecan sheddase (33, 34). Therefore, HPSE (in)directly contributes to increased HS and syndecan-1 plasma levels, which are both markers of endothelial glycocalyx degradation. The amount of HS in plasma of acute dengue patients (n=30) was significantly increased in acute dengue compared to convalescent dengue and healthy controls (**Figure 3A**). Furthermore, HS plasma levels were assessed over time (n=10), which revealed that HS plasma levels are highest at hospital admission and restore to normal levels during the following days (**Figure 3B**). Similarly, syndecan-1 plasma levels (n=30) were increased in acute dengue compared to convalescent dengue (**Figure 3C**), and highest at hospital admission, whereafter they decreased to normal levels (n=10) (**Figure 3D**). Although only syndecan-1 (**Figure 3F**) and not HS (**Figure 3E**) correlated with HPSE activity at day 1 upon hospital admission, a strong and significant correlation could be observed for both HS (**Figure 3G**) and syndecan-1 (**Figure 3H**) over time. In summary, degradation of the endothelial glycocalyx is observed in acute dengue patients, which most likely is mediated by increased HPSE activity.

### Increased Plasma HPSE Activity Associates With Platelet Number and Might be Supported by Thrombin or DENV2-Induced HPSE Release From Platelets

**Figure 4:**
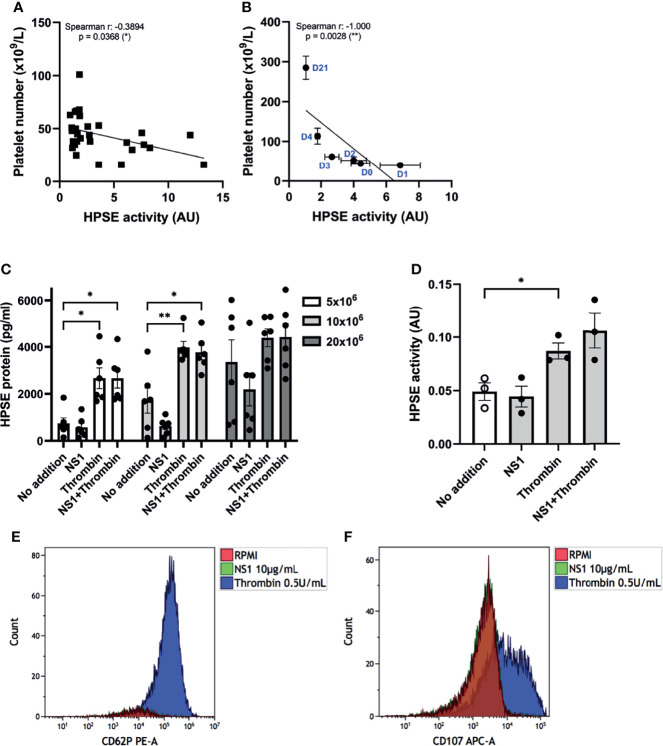
Platelets activation and HPSE activity in dengue patients. **(A)** Correlation between HPSE activity and platelet number of acute dengue patients at day 1 upon hospitalization (n=30). **(B)** Correlation between HPSE activity and platelet number of acute dengue patients over time (n=10). For each time point the mean and SEM HPSE activity/platelet number of all patients measured at that time point is depicted. **(C)** Released HPSE protein from platelet-rich plasma (PRP) of healthy volunteers (N = 6) pretreated with 10 µg/mL DENV NS1 or RPMI for 20 minutes, and subsequently incubated with 0.5 U/mL thrombin or RPMI for another 20 minutes. **(D)** HPSE activity in the PRP supernatant (n=3). **(E)** Expression of p-selectin (CD62P) or **(F)** lysosomal LAMP-1 (CD107) in platelets after 20 minutes incubation with DENV NS1 or thrombin as measured by flow cytometry. NS1 stimulated platelet flow cytometry plots exactly overlay no RPMI platelets plots. The flow cytometry data shown is from one donor but has been repeated with at least three donors. Data were presented as mean ± SEM and tested for normal distribution with D’Agostino & Pearson omnibus normality test. Statistical differences were calculated using one-way ANOVA followed by Turkey’s multiple comparisons test. Correlation analysis was performed with Spearman’s correlation coëfficient (*p < 0.05, **p < 0.01). HPSE, heparanase; NS1, DENV NS1; AU, arbitrary units.

Platelets store relatively high amounts of HPSE in their dense-granules and lysosomes, and are known to release HPSE upon activation, specifically by thrombin stimulation (21). Remarkably, a recent study suggested the increase of HPSE mRNA expression as well as enzymatic activity in platelets of sepsis patients, which was associated with sepsis-associated mortality (35). Since platelet activation, as well as coagulation activation are prominent features of dengue, we further explored the possible role of platelets to the observed increased plasma HPSE activity and presence of endothelial glycocalyx degradation products in plasma from dengue patients. Plasma HPSE activity inversely correlated with platelet number (**Figure 4A**) at day one after study enrollment, which was the day with the lowest mean platelet count. Moreover, platelet number showed a strong and significant correlation with HPSE activity over time (**Figure 4B**).

Next, we investigated whether DENV2 NS1 in the presence or absence of thrombin induces HPSE release from platelets. The platelet number in the well determined the level of released HPSE (**Figure 4C**). Incubation with thrombin enhanced the HPSE release (**Figure 4C**) and HPSE activity (**Figure 4D**) from platelets, whereas incubation with DENV2 NS1 had no significant effect. Pretreatment of platelets with DENV2 NS1 and subsequent incubation with thrombin did not further enhance HPSE secretion or activity (**Figures 4C, D**). We also evaluated platelet activation by measuring cell surface expression of P-selectin (CD62P) and the lysosomal LAMP-1 (CD107a), which confirmed that only thrombin is able to induce CD62P and CD107a expression, whereas (pre)incubation with NS1 did not induce CD62P and CD107a expression (**Figures 4E, F**).

Finally, we incubated platelets of healthy donors with live whole DENV2 virus. The DENV2 virus induced HPSE release from platelets comparable to thrombin within 20 minutes of incubation (**Supplementary Figure 3A**). Interestingly, this HPSE release was in conjunction with the disappearance of platelets as measured by CD61 (**Supplementary Figure 3B**), which might suggest that platelet fragmentation or aggregation occurs; thus making it impossible to measure the platelet activation with flow cytometry analysis. Taken together, HPSE release upon thrombin or DENV2 virus exposure may contribute to the increased plasma HPSE activity in dengue patients.

## Discussion

In this study we demonstrate the increase of plasma HPSE activity in hospitalized patients with acute dengue virus infection and further explore its relationships with markers of plasma leakage, endothelial glycocalyx degradation, albuminuria and bleeding manifestations. We also evaluate the possible role of platelet activation to the increased plasma HPSE activity in dengue.

The pathogenesis of plasma leakage in dengue is complex and still incompletely understood. Disruption of the endothelial glycocalyx is now considered to play a central role in the transient endothelial hyper-permeability (15, 36). HPSE is a critical component of the glycocalyx function and has been linked to various diseases characterized by inflammation and plasma leakage such as sepsis, proteinuric kidney diseases and viral diseases like COVID-19 (12, 17, 18, 31, 35, 37–39). In line with our current findings, several *ex vivo* and *in vitro* studies have previously explored the role of HPSE protein in dengue (15, 16, 35). Using an activity assay, we now provide additional proof that the circulating plasma HPSE is indeed active, and we identified associations between plasma HPSE activity and markers of plasma leakage, endothelial glycocalyx disruption and bleeding. Therefore, our present data strengthen the notion that increased plasma HPSE activity is an important mediator of endothelial glycocalyx disruption and plasma leakage in patients with dengue virus infection.

Previous clinical studies in dengue patients have described the increase of endothelial glycocalyx degradation products such as heparan sulfate, hyaluronic acid and shedded syndecan-1 in plasma (11, 16). A recent *in vitro* study also showed that DENV NS1 induces endothelial glycocalyx layer degradation, thereby promoting a cytokine-independent endothelial permeability (40). In line with these previous findings, our current data further provide proof by establishing relationships between increased plasma HPSE activity and endothelial glycocalyx degradation markers in acute dengue. Notably, we recently also showed a possible role of HPSE in mediating endothelial glycocalyx degradation in severe coronavirus disease (COVID-19) associated lung edema (31). Pleural effusion, which is observed in a subgroup of COVID-19 patients (41), is a prominent feature of plasma leakage in dengue patients as well (42). Moreover, HPSE is crucial for the development of glomerulonephritis and diabetic nephropathy (17, 18) as HPSE knock-out mice disease models showed significantly reduced proteinuria, which is another feature of dengue-associated plasma leakage (19). We now reveal that the urinary HPSE of acute dengue patients is active, which may associate with proteinuria in dengue patients.

We provide further evidence for a role of HPSE-mediated degradation of the glycocalyx in acute dengue. However, other studies propose different mediators of endothelial glycocalyx degradation leading to endothelial barrier dysfunction and plasma leakage. For instance, patients with dengue hemorrhagic fever have increased levels of neutrophil extracellular traps (NETs) (43, 44). NETs are web-like chromatin structures released by neutrophils upon exposure to various stimulants and contribute to endothelial damage, inflammation and increased vascular permeability (45, 46). Another group suggested that tryptase, a mast cell derived protease, promotes vascular leakage through break-down of endothelial cell tight junctions (47). Interestingly, both NETs and tryptase are known to (in)directly induce shedding of the endothelial glycocalyx (45, 48). However, it could well be that a combination of mediators initiate the substantial vascular leakage observed in dengue.

HPSE is secreted by various cells including circulating blood cells such as leukocytes and platelets. Platelets are known to play a crucial role in maintaining vascular integrity (24) during dengue and modulate leukocyte diapedesis through HPSE secretion (25). Furthermore, DENV NS1 has been shown to initiate an inflammatory response through TLR4 on monocytes and platelets, and also induces the release of cathepsin L, which is crucial for the formation of active HPSE (49, 50). Whereas platelets on the one hand may preserve endothelial integrity, release of HPSE from platelets in pathological conditions may increase plasma leakage. In line with our current finding, a recent report showed that intra-platelet HPSE mRNA expression in dengue patients is increased (35). Different mechanisms may contribute to the link between platelet activation, plasma leakage and increased HPSE activity in dengue. First, platelet lysosomes contain multiple bioactive molecules including HPSE, which can be released upon platelet activation. Second, the release of p-selectin (CD62p) from platelet granules is coherent with upregulation of platelet HPSE activity (20). Third, multiple circulating biological mediators in plasma of dengue patients might contribute to platelet activation in dengue. In our study, we demonstrated that thrombin and DENV2 virus, but not recombinant DENV2 NS1, induced the HPSE release. Intriguingly, in contrast with previous reports on platelet activation upon NS1 incubation (50, 51), we could not show NS1-induced platelet activation or NS1-induced HPSE release by platelets. Some studies suggest that the specific receptor for DENV NS1 on platelet is TLR4, a ligand for LPS (50), while another study showed that TLR4 stimulation did not induce classical platelet activation upon LPS stimulation (52). Furthermore, Quirino-Teixeira et al. could not induce platelet lysosomal release after 3 hours incubation with DENV NS1 (53). The contradicting findings on the role of DENV NS1 in activating platelets may be due to differences in biological activity of the NS1 preparations used in different studies, and/or differences in purification protocols for platelets. Nevertheless, with thrombin stimulation we could show both classical platelet activation and release of HPSE protein and activity, which is in line with a previous study that demonstrated release of HPSE upon thrombin stimulation by platelets, but also granulocytes and mono-nuclear cells (21). Notably, a different pattern of platelet activation has been suggested upon thrombin stimulation in which platelets fully fragment causing maximal release of its contents (54). DENV2 virus is also known to induce both platelet activation and apoptosis (22). We demonstrated that platelets release HPSE protein upon DENV2 virus exposure. Although we focused on platelets in the current study, we cannot exclude other sources of HPSE in dengue, which may include immune cells as well as endothelial cells. Altogether, our data suggest that thrombin and DENV2 virus, but not DENV NS1, might contribute to the increased plasma HPSE activity in acute dengue patients.

In summary, our study shows that plasma HPSE activity as well as the endothelial glycocalyx degradation products, HS and syndecan-1, are increased in acute dengue patients. Moreover, HPSE activity was associated with disease severity and several clinical markers of plasma leakage, thereby strengthening the possibility that HPSE may be key for initiating the vascular leakage observed in dengue. Furthermore, our data propose that the observed increased HPSE activity might be released from thrombin or DENV2-stimulated platelets. Inhibition of HPSE might therefore prove a treatment option to prevent disruption of the endothelial glycocalyx barrier function in dengue. Heparins are known to competitively inhibit HPSE (37) and we recently showed that use of low molecular weight heparins in prophylactic dose in non-ICU patients with COVID-19 was associated with a reduced HPSE activity (31). One study in dengue patients showed that heparin accelerated restoration of platelet numbers (55). Further studies are required to explore to possible role of heparins, and preferably modified heparinoids without anticoagulation activity, as a possible adjunctive treatment for patients with dengue viral infection.

## Data Availability Statement

The raw data supporting the conclusions of this article will be made available by the authors, without undue reservation.

## Ethics Statement

The clinical trial was approved by the Medical Research Ethics Committee, Faculty of Medicine, Diponegoro University, Semarang and Faculty of Medicine, Universitas Padjadjaran, Bandung, Indonesia with reference number No:650/EC/FK-RSDK/XI/2017. For *ex vivo* experiments with healthy volunteers, ethical approval was provided by Medical Research Ethics Committee Arnhem-Nijmegen Region (NL32357.091.10). Written informed consents were obtained from all subjects.

## Author Contributions

BB, FG, GO, RR, QM, and JV designed the experiments. BB, FG, RA, QM, and JV analyzed the data and wrote the manuscript. BB, FG, and GO performed the experiments. SR, SP, RT, MG, BA, and QM provided the patient samples, clinical data and performed the ultrasonography examinations. QM and JV supervised the study. All authors critically reviewed and edited the manuscript. All authors contributed to the article and approved the submitted version.

## Funding

BB was supported by the Radboud Institute of Molecular Life Sciences (RIMLS) PhD-program. FG is financially supported by the Indonesian Endowment Fund for Education (LPDP) Scholarship from the Ministry of Finance Republic of Indonesia. Patient inclusion was financially supported by ZonMW (grant 451001005). The funders had no role in study design, data collection and analysis, decision to publish, or preparation of the manuscript.

## Conflict of Interest

The authors declare that the research was conducted in the absence of any commercial or financial relationships that could be construed as a potential conflict of interest.

## Publisher’s Note

All claims expressed in this article are solely those of the authors and do not necessarily represent those of their affiliated organizations, or those of the publisher, the editors and the reviewers. Any product that may be evaluated in this article, or claim that may be made by its manufacturer, is not guaranteed or endorsed by the publisher.

## Supplementary Material

Supplementary Figure 1 Relationship of HPSE activity with hematocrit concentration and plasma albumin level as clinical parameters of plasma leakage. Correlations of HPSE activity in plasma at day of admission (day 0) and day 1, 2 and 3 with **(A)**. hematocrit concentration and **(B)**. plasma albumin level. Data are presented as individual values for each patient and correlation analysis was performed with Spearman’s correlation coëfficient (*p < 0.05, **p < 0.01). HPSE, heparanase; AU, arbitrary units.

Supplementary Figure 2 Relationship of HPSE activity with gall-bladder wall thickness and albumin-creatinine ratio as clinical parameters of plasma leakage. Correlations of **(A)**. HPSE activity in plasma at day of admission (day 0) and day 1, 2 and 3 with gall-bladder wall thickness and **(B)**. urinary HPSE activity at day of admission (day 0) and day 1, 2 and 3 with albumin-creatinine ratio. Data are presented as individual values for each patient and correlation analysis was performed with Spearman’s correlation coëfficient (*p < 0.05, ***p < 0.01). HPSE, heparanase; AU, arbitrary units; ACR, albumin-creatinine ratio.

Supplementary Figure 3 Platelet stimulation with DENV2 induces release of HPSE protein. Platelet-rich plasma (2x10^6^/mL) from adult healthy volunteers (n=3) were stimulated with DENV2 (MOI 1.6) or thrombin (0.5 U/mL) for 20 minutes whereupon **(A).** HPSE protein release from the platelets was measured in the supernatant and **(B).** Expression of integrin beta-3 (CD61) platelet marker was measured by flow cytometry from three donors in duplo. Data were presented as mean ± SEM and tested for normal distribution with D’Agostino & Pearson omnibus normality test. Statistical differences were calculated using one-way ANOVA followed by Turkey’s multiple comparisons test (*p < 0.05, **p < 0.01). HPSE, heparanase.
