# Supplementary figures and images for: Increased Plasma Heparanase Activity and Endothelial Glycocalyx Degradation in Dengue Patients Is Associated With Plasma Leakage
Source: Front Immunol. 2021 Dec 20;12:759570. doi: 10.3389/fimmu.2021.759570 (PMC8722520; doi:10.3389/fimmu.2021.759570)

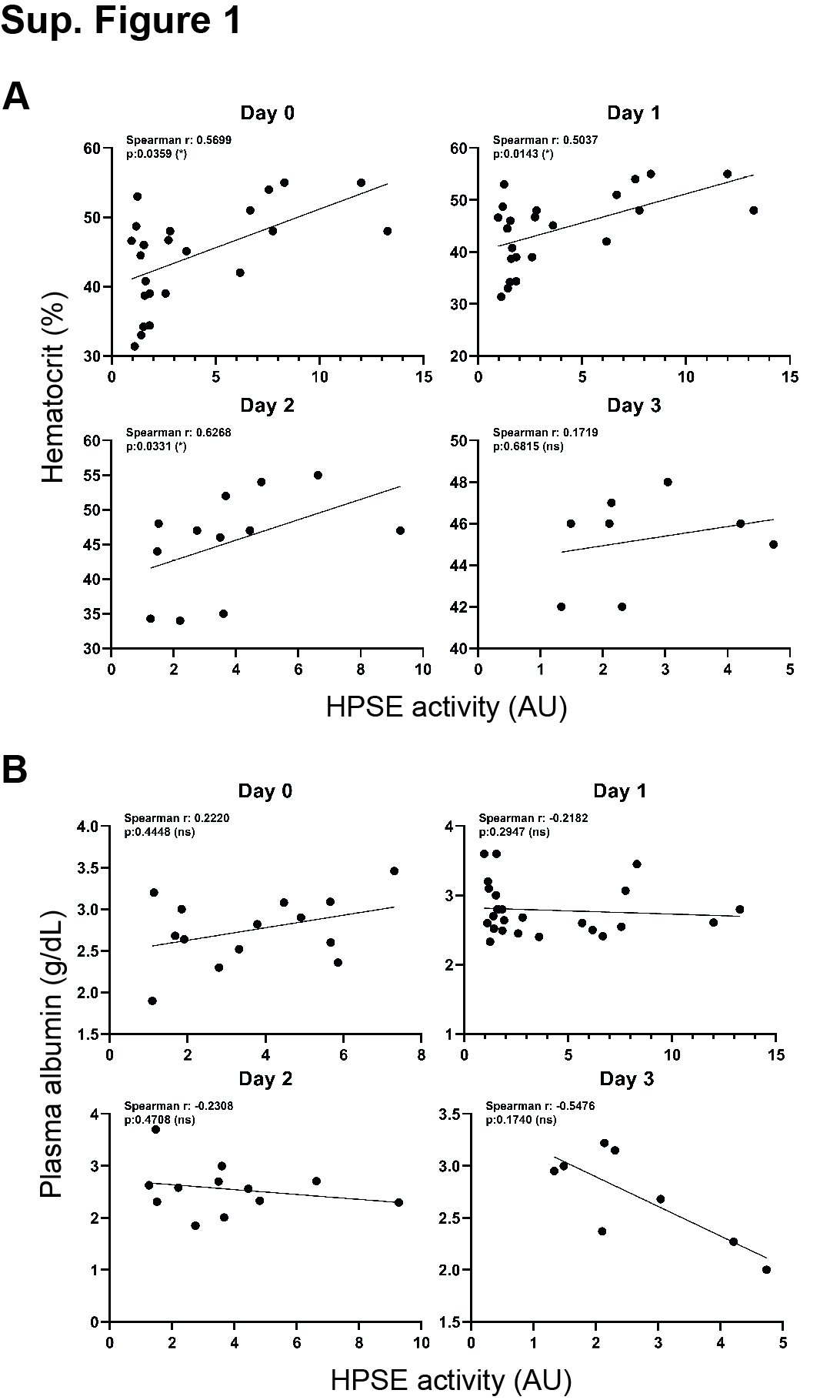

Supplement: Supplementary Figure 1 — Relationship of HPSE activity with hematocrit concentration and plasma albumin level as clinical parameters of plasma leakage. Correlations of HPSE activity in plasma at day of admission (day 0) and day 1, 2 and 3 with (A). hematocrit concentration and (B). plasma albumin level. Data are presented as individual values for each patient and correlation analysis was performed with Spearman’s correlation coëfficient (*p < 0.05, **p < 0.01). HPSE, heparanase; AU, arbitrary units. [file Image_1.tif]

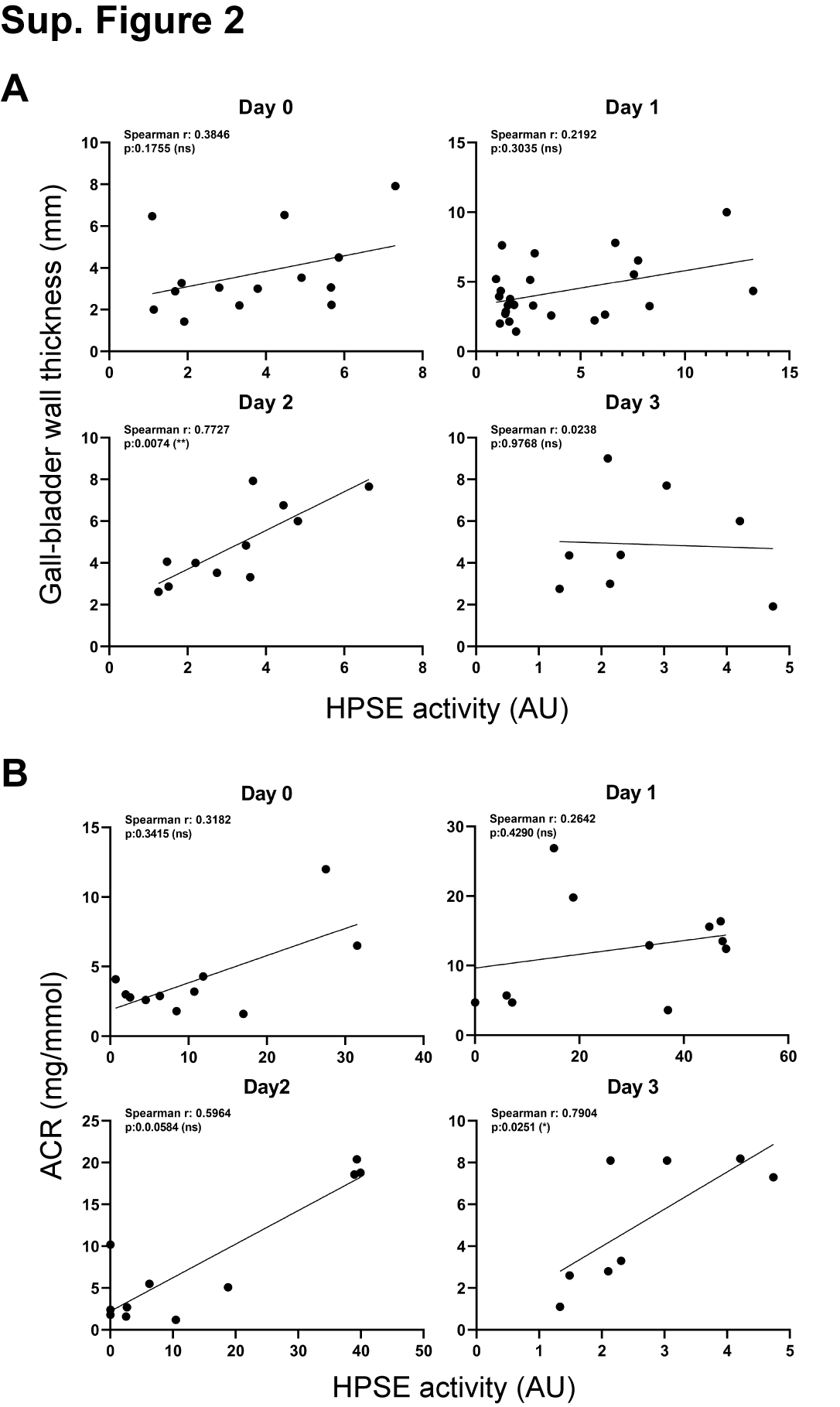

Supplement: Supplementary Figure 2 — Relationship of HPSE activity with gall-bladder wall thickness and albumin-creatinine ratio as clinical parameters of plasma leakage. Correlations of (A). HPSE activity in plasma at day of admission (day 0) and day 1, 2 and 3 with gall-bladder wall thickness and (B). urinary HPSE activity at day of admission (day 0) and day 1, 2 and 3 with albumin-creatinine ratio. Data are presented as individual values for each patient and correlation analysis was performed with Spearman’s correlation coëfficient (*p < 0.05, ***p < 0.01). HPSE, heparanase; AU, arbitrary units; ACR, albumin-creatinine ratio. [file Image_2.tif]

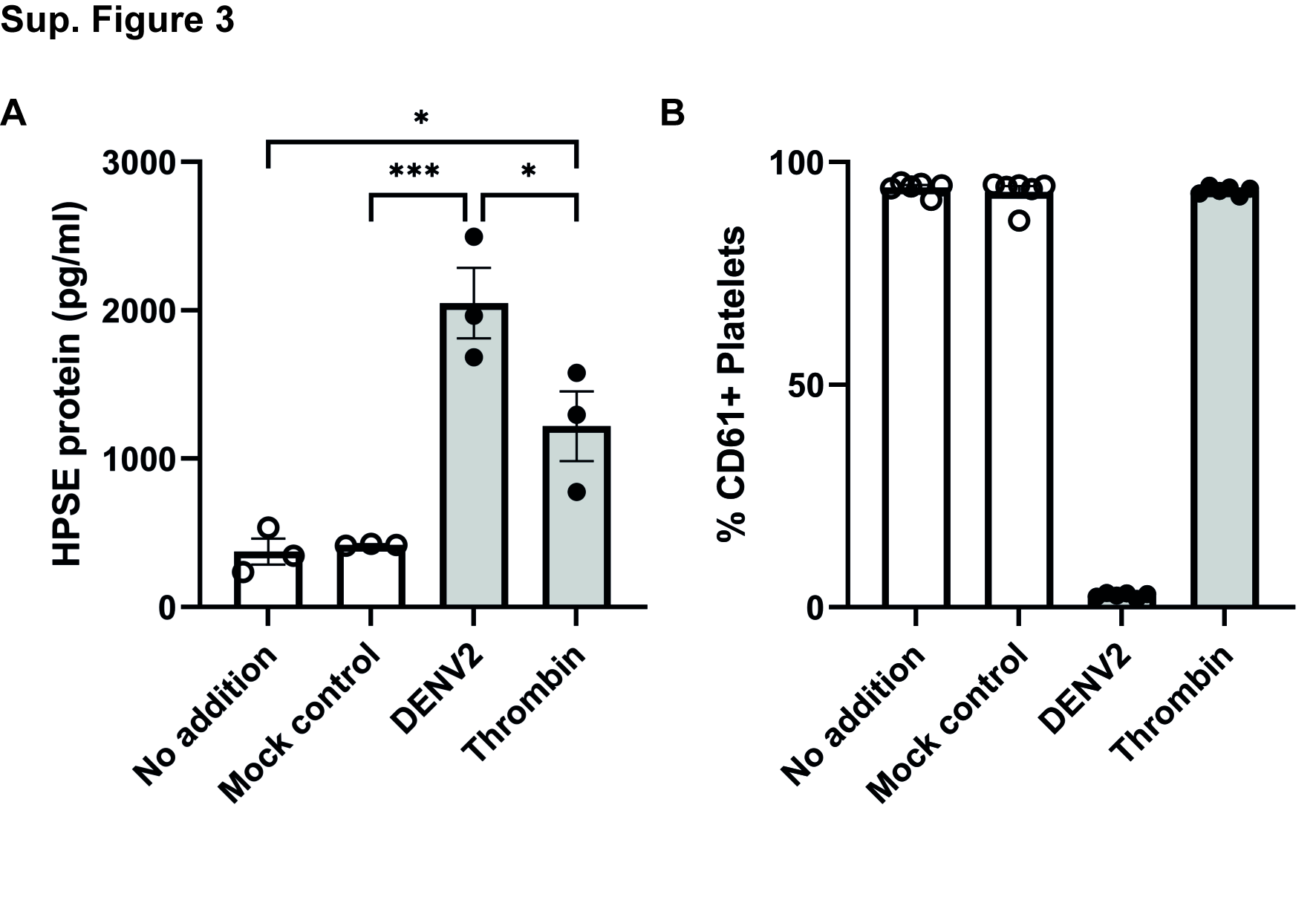

Supplement: Supplementary Figure 3 — Platelet stimulation with DENV2 induces release of HPSE protein. Platelet-rich plasma (2x10^6^/mL) from adult healthy volunteers (n=3) were stimulated with DENV2 (MOI 1.6) or thrombin (0.5 U/mL) for 20 minutes whereupon (A). HPSE protein release from the platelets was measured in the supernatant and (B). Expression of integrin beta-3 (CD61) platelet marker was measured by flow cytometry from three donors in duplo. Data were presented as mean ± SEM and tested for normal distribution with D’Agostino & Pearson omnibus normality test. Statistical differences were calculated using one-way ANOVA followed by Turkey’s multiple comparisons test (*p < 0.05, **p < 0.01). HPSE, heparanase. [file Image_3.tif]
